# Sustainable Dentistry: A Comprehensive Review of the Recycling Techniques for Gypsum Products in Prosthodontics

**DOI:** 10.7759/cureus.55997

**Published:** 2024-03-12

**Authors:** Shubham U Tawade, Mithilesh M Dhamande, Surekha A Dubey, Seema Sathe, Madhavi S Selukar, Ankita Pathak

**Affiliations:** 1 Prosthodontics, Sharad Pawar Dental College and Hospital, Datta Meghe Institute of Higher Education & Research, Wardha, IND; 2 Prosthodontics and Crown & Bridge, Sharad Pawar Dental College and Hospital, Datta Meghe Institute of Higher Education & Research, Wardha, IND

**Keywords:** eco-friendly practices, dental waste management, environmental impact, prosthodontics, gypsum recycling, sustainable dentistry

## Abstract

This review explores the pivotal role of sustainable dentistry with a specific focus on the recycling of gypsum products in prosthodontics. As oral health practices increasingly impact the environment, the adoption of sustainable approaches becomes imperative. The review delves into the environmental challenges posed by gypsum waste in prosthodontics and examines current recycling techniques, presenting key findings and successful case studies. The call to action is directed towards the dental community, urging practitioners, educators, and policymakers to prioritize sustainable practices, encompassing responsible waste management and the incorporation of eco-friendly materials. Looking to the future, the review envisions a promising landscape for sustainable dentistry in prosthodontics, propelled by emerging technologies and a collective commitment to environmentally conscious oral healthcare. Ultimately, this review serves as a catalyst for positive change, advocating for a transformative shift toward sustainability within the dental community.

## Introduction and background

Recycling in dentistry plays a pivotal role in maintaining sustainable practices, aiming to mitigate the environmental footprint associated with dental procedures. The traditional disposal methods often involve the incineration or landfilling of waste materials, contributing to pollution and resource depletion. Recycling, on the other hand, presents a viable alternative, promoting the reuse of materials, reducing the need for raw resources, and minimizing waste generation. This not only aligns with environmental conservation goals but also contributes to the ethical and social responsibility of dental practitioners [1].

Prosthodontics, a specialized branch of dentistry focusing on the restoration and replacement of teeth, heavily relies on gypsum products for various applications, including dental impressions and prosthetic models. Gypsum, while indispensable in prosthodontics, poses environmental challenges due to the issues associated with its disposal. The gypsum waste generated in dental practices, if not managed sustainably, can contribute to environmental degradation. Hence, understanding and implementing recycling strategies for gypsum products in prosthodontics is crucial for achieving a more sustainable dental industry [2].

The primary purpose of this review is to provide a comprehensive examination of the recycling of gypsum products in prosthodontics within the context of sustainable dentistry. By exploring the current state of gypsum recycling techniques, identifying challenges, and presenting case studies of successful implementation, this review aims to provide valuable insights to dental professionals, researchers, and policymakers. Ultimately, the goal is to foster a greater understanding of sustainable practices in prosthodontics and inspire positive changes in the broader landscape of dental care.

## Review

Overview of gypsum products in prosthodontics

Gypsum products are vital in prosthodontics, offering versatile applications across various dental procedures. These materials are indispensable for creating models, casts, and dies, which form the foundation for fabricating dental appliances and restorations. Several types of gypsum products are commonly employed within the field of dentistry. Dental stone, categorized as Type IV gypsum, serves as a high-strength material crucial for producing models intended for fixed and combined prostheses [3]. Plaster of Paris, a finely ground gypsum powder, finds widespread use in dental offices and laboratories for crafting models and casts [4]. Gypsum investment material, another essential component, is used to produce dental casts mixed with water to form either a fluid or stable mass [4]. On the other hand, dental plaster plays a significant role in finalizing removable prostheses, particularly following the registration of vertical and centric relations and fitting trials [3].

**Figure 1:**
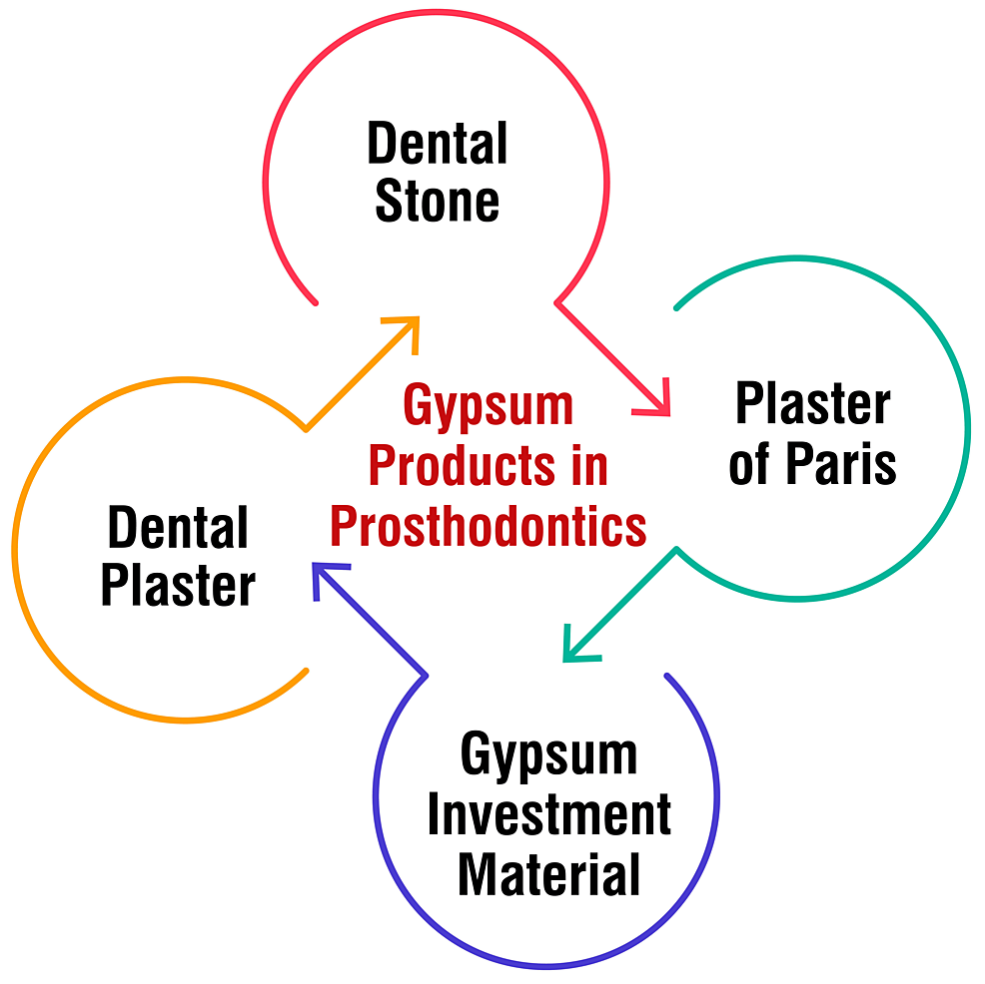
Gypsum products in prosthodontics Figure created by the author

These gypsum products typically come in fine powders, necessitating mixing with water to achieve the desired consistency, whether as a fluid or stable mass. The precise ratios of powder to water are critical factors influencing the strength and hardness of the resultant cast or model. Minimizing the mixture's water content enhances the final product's strength [5]. Despite their utility, gypsum products contribute to waste generation in dental settings. Fortunately, gypsum waste, comprising materials such as dental stone, plaster of Paris, gypsum investment material, and dental plaster, can be effectively recycled without requiring extensive chemical or physical modification. Various recycling methods have been devised, including utilizing a 20% ammonium bicarbonate solution, semi-dry pressing technology, and calcination [6,7]. Proper disposal practices are also emphasized, with guidelines stipulating that gypsum waste should be segregated from other types of waste and either disposed of in a dedicated cell within a landfill or directed to specialized gypsum recycling services [8]. Gypsum products in prosthodontics are shown in Figure 1.

Environmental impact of traditional disposal methods

The conventional methods of disposing of gypsum waste, including storage, landfilling, and discarding, carry considerable environmental consequences. When gypsum is deposited in landfills alongside other waste materials, it has the potential to decompose, releasing hydrogen sulfide, a noxious gas known for its toxic properties and foul odor, posing serious threats to public health and safety [8]. Moreover, gypsum in landfills fosters conditions conducive to generating methane, a potent greenhouse gas, due to its carbon-containing nature [8]. Furthermore, the disposal of gypsum waste in landfills exacerbates the strain on landfill sites and escalates associated costs [9]. In response to these environmental challenges, there is a growing recognition of gypsum recycling as a more sustainable alternative. Recycling gypsum waste can diminish reliance on quarrying and the production of virgin gypsum, reducing energy consumption and greenhouse gas emissions [8]. In the dental context, exploration into methods like calcination for recycling dental gypsum waste has emerged to alleviate its environmental footprint [7]. Given the adverse environmental implications associated with the conventional disposal of gypsum waste, including releasing toxic gases and escalated landfilling costs, promoting gypsum recycling is a more sustainable approach to mitigate its environmental impact.

The need for sustainable practices in dentistry

The imperative for sustainable practices within dentistry has surged in relevance owing to the environmental repercussions of conventional dental procedures. Recognizing this urgency, the FDI World Dental Federation has underscored the necessity for sustainability in oral healthcare and has issued guidelines to guide oral health professionals. These guidelines advocate for reducing energy, water, paper, and environmentally harmful materials consumption, alongside minimizing air emissions and water releases [10]. Concurrently, there is a push for dental materials companies to assess and alleviate the environmental footprint of their products, particularly concerning the generation of harmful byproducts [11]. Of particular concern is the traditional disposal of gypsum waste, widely utilized in dental practices, which poses notable environmental risks, including the emission of toxic gases and heightened landfilling costs [10]. Consequently, promoting gypsum waste recycling emerges as a paramount solution to mitigate its adverse environmental impact [7,12]. Dental establishments can enact several sustainable measures, including implementing eco-friendly sterilization protocols, installation of amalgam separators, cessation of aerosol product usage, and strategic office layout planning to optimize natural light and ventilation [13]. Furthermore, dental professionals can augment their knowledge base through educational initiatives like the Sustainable Dentistry course provided by the Centre for Sustainable Healthcare to gain deeper insights into sustainable dental practices [11].

Gypsum recycling techniques

Gypsum Composition and Properties

Gypsum recycling constitutes a process whereby gypsum waste originating from construction sites is transformed into recycled gypsum, serving as a substitute for virgin gypsum raw materials in manufacturing processes. This transformative cycle renders gypsum fully recyclable, perpetually generating a raw material capable of replicating the same product. Notably, the environmental benefits of gypsum recycling are underscored by statistics indicating that recycling one metric ton of gypsum can conserve 28 kWh of energy and 4 pounds of aluminum [4]. The recycling process typically commences at recycling centers, where construction site waste undergoes separation, with the paper being isolated from gypsum. Subsequently, the gypsum is finely pulverized into a powder, rendering it suitable for reuse. This recycled gypsum finds application across various products, offering an environmentally sound alternative to landfill disposal and curbing waste at its source [14].

Recycled gypsum boasts a diverse array of advantageous uses, including its utilization as a soil amendment, incorporation into cement and specialty ready-mix concrete, and integration into an assortment of products such as plastics, adhesives, sealants, and specialty cement. Furthermore, recycled gypsum enhances fire retardancy, tensile strength, and translucency of polymers. By harnessing recycled gypsum, natural resources are conserved, landfill volumes are reduced, and avenues for environmental preservation and site revitalization are fostered [15]. In the dental realm, the recycling of gypsum waste encompassing materials like dental stone, plaster of Paris, gypsum investment material, and dental plaster can be achieved without necessitating chemical or physical alterations. Various recycling methodologies are viable, from employing gypsum waste as a plant compost additive or nutrient source to employing chemical and physical techniques. Recycling gypsum waste is an indispensable approach for managing it, as it enables the disinfection and sterilization necessary for its reuse in industrial and other applications [6].

Review of Existing Recycling Methods

Mechanical processes: Gypsum recycling often employs mechanical processes involving steps like grinding to reduce waste and eliminate impurities and sieving and pressing to enhance the material's quality and usability [16]. This approach effectively reduces waste at its source, yielding recycled gypsum suitable for diverse applications like concrete additives, plaster, stucco, compost, and paper products [14,17]. Innovative techniques such as semi-dry pressing technology show promise in gypsum waste recycling, offering a sustainable solution for repurposing discarded materials [18]. Moreover, chemical processing methods provide an alternative avenue, allowing for the reuse of gypsum in construction materials, thereby diverting it from landfills [19]. These diverse recycling methods highlight the potential for sustainable gypsum waste management across industries, including dentistry and construction.

Chemical processes: Chemical methods also play a crucial role in gypsum waste recycling. Specifically, developed chemical processes enable the reclamation of gypsum from building materials, preventing its disposal in landfills [19]. Notably, research on dental gypsum recyclability through calcination, involving high-temperature heating to eliminate impurities, offers another avenue for sustainable waste management [7]. However, due to its potentially hazardous properties, it is imperative to handle dental gypsum waste with care, segregating it from other waste streams [7]. Chemical processes present a viable approach to recycling gypsum waste, thus reducing its environmental footprint.

Biological processes: Biological pathways provide additional avenues for recycling gypsum waste across various applications. For instance, the reclamation of flue gas desulfurization (FGD) gypsum utilizing mixed sulfate-reducing bacteria (SRB) with economical carbon sources like sewage digest or synthesis gas showcases the potential of biological methods [20]. Through an anaerobically digested municipal sewage sludge (AD-MSS) medium, SRBs demonstrate efficient FGD gypsum reduction rates, with subsequent processes yielding elemental sulfur recovery and high chemical oxygen demand utilization [20]. Similarly, dental gypsum's calcination-based recyclability underscores biological processes' applicability in waste management [7]. By leveraging these biological approaches, gypsum waste can be repurposed sustainably, spanning industries such as FGD gypsum reclamation and dental gypsum recycling, thereby mitigating environmental impact.

Challenges and Opportunities in Gypsum Recycling

Gypsum recycling presents both challenges and opportunities within the industry. While it poses complexities, the growing demand for eco-friendly products and the advantages of recycling gypsum, such as decreased power consumption and minimal emissions, create significant growth prospects, particularly for manufacturers [21]. Notably, the construction sector sees a primary opportunity in gypsum board recycling, as it offers a fully recyclable alternative that can replace waste management expenses with value-added products [21]. However, challenges persist, including the intricate and costly recycling process, the necessity for precise source separation during demolition, and the imperative of developing robust recycling infrastructure to meet escalating demands [22].

Gypsum waste holds potential as a recyclable additive for concrete, plaster, stucco, soil amendment, and various industrial applications, such as water treatment and animal bedding [17]. Employing diverse methods like ammonium bicarbonate solution, semi-dry pressing technology, and calcination facilitates gypsum recycling [18,23]. Despite challenges such as specific drying requirements and potential build-up during processing, these hurdles can be overcome through rigorous testing and optimization of processing techniques [24]. Moreover, gypsum recycling in dentistry presents opportunities to minimize environmental impact and enhance the availability of prosthetic and orthotic applications [23]. While gypsum recycling encounters obstacles, the industry witnesses growth and heightened interest, driven by endeavors to devise sustainable and eco-friendly disposal procedures [22].

Case studies

Successful Implementation of Gypsum Recycling in Dental Practices

Numerous case studies highlight the successful integration of gypsum recycling practices within dental settings. One notable study delved into the recyclability of dental gypsum through calcination, a method involving the application of heat to eliminate water and carbon dioxide. This approach has proven effective in recycling gypsum waste, curtailing its environmental impact [7]. Dental establishments can avail themselves of gypsum recycling services to responsibly manage their waste, ensuring its collection and disposal in an eco-friendly manner [8]. Another impactful initiative aimed at minimizing gypsum waste entails the adoption of reusable and sterilizable impression trays in dental practices. Practices can substantially mitigate waste generation by opting for such trays over disposable counterparts [12]. Furthermore, a study conducted among Peruvian undergraduate dental students evaluated their comprehension and awareness regarding effective dental materials recycling and waste management. Results indicated a noteworthy proportion of students were cognizant of the recyclability of gypsum and other dental materials [25]. Moreover, research has validated that recycled gypsum plaster exhibits comparable working properties to virgin gypsum plaster, rendering it suitable for diverse applications, including mold manufacturing [23]. These case studies underscore the feasibility of implementing gypsum recycling practices in dental environments, fostering a more sustainable and environmentally conscious approach to waste management.

Environmental and Economic Benefits

Multiple case studies have underscored the dual environmental and economic advantages of gypsum recycling. By diverting gypsum waste from traditional disposal routes, recycling initiatives contribute to pollution reduction, solid waste minimization, and decreased energy and water usage, consequently alleviating waste management expenses [14,26]. Furthermore, using recycled gypsum as a fertilizer and soil amendment emerges as a pivotal strategy for enhancing sustainability in agriculture. This application promotes water infiltration and reintroduces vital nutrients like calcium and sulfur into the soil, fostering sustainable crop production practices [14,15]. Additionally, adopting reusable and sterilizable impression trays in dental practices represents a tangible measure to diminish reliance on disposable gypsum trays, thereby curbing waste generation [15]. Moreover, cultivating knowledge and awareness among dental students regarding effective dental materials recycling and waste management is crucial in facilitating the successful integration of gypsum recycling practices within dental settings [25]. Furthermore, gypsum recycling presents an attractive prospect for businesses, as it addresses disposal challenges and transforms an environmental concern into a viable business opportunity [14]. Overall, the multifaceted benefits of gypsum recycling highlight its significance as an environmentally sustainable and economically viable alternative, offering a win-win solution for both environmental stewardship and financial prudence.

Lessons Learned and Best Practices

Multiple case studies have delineated key best practices and valuable lessons for effective gypsum recycling. Foremost among these is the on-site segregation of gypsum waste, a foundational practice essential for fostering a circular economy for gypsum products [27]. Notably, developing Best Practice Indicators (BPIs) for gypsum recycling has provided a framework focusing on deconstruction, recycling, and reincorporation processes, offering guidance for optimizing recycling practices [28]. Furthermore, adopting reusable and sterilizable impression trays is a practical measure to diminish reliance on disposable gypsum trays, thereby mitigating waste generation [29]. Additionally, fostering knowledge and awareness among dental students regarding effective dental materials recycling and waste management is pivotal in facilitating the successful integration of gypsum recycling practices within dental settings [25]. Moreover, successful gypsum recycling hinges on meticulous attention to source separation, necessitating deliberate efforts by contractors at construction and demolition sites to segregate gypsum waste from other waste streams [22]. Additionally, dental practices must establish a comprehensive Waste Management Policy to ensure proper disposal of gypsum waste [30]. These case studies underscore that gypsum recycling can potentially deliver both environmental and economic benefits, underscoring the critical importance of implementing sound waste management practices for successful gypsum recycling initiatives.

Current Regulations on Dental Waste Management

The regulatory framework governing dental waste management encompasses stringent guidelines to ensure the safe disposal of gypsum waste. Under the Environmental Protection Act of 1990, all waste producers must adhere to a Duty of Care, necessitating the proper and correct disposal of waste materials [8]. Dental clinics, laboratories, and surgeries must comply with established protocols governing the safe disposal of dental waste, which have been meticulously documented [8]. Notably, waste originating from dentistry may be classified as toxic if not handled with specificity and segregation from other waste streams [7]. Regarding disposal options for gypsum waste, dental practices typically have two main avenues: gypsum recycling and landfilling in designated cells within a landfill specifically allocated for such waste [8].

To ensure compliance with regulatory standards, dental establishments must institute procedures for identifying and segregating gypsum waste, thereby facilitating its appropriate disposal [8]. Additionally, it is imperative for dental practices to review and update their Waste Management Policy to align with regulatory requirements, as failure to do so may lead to scrutiny from local authority waste enforcement officers, who have the authority to inspect various forms of waste, including hazardous, general, and recycling waste [30]. Moreover, developing Best Practice Indicators (BPIs) specific to gypsum recycling underscores the importance of adhering to best practices throughout the deconstruction, recycling, and reincorporation processes [30]. By adhering to these regulations and guidelines, dental practices can ensure the safe and responsible disposal of gypsum waste, mitigating environmental risks and upholding regulatory compliance standards.

Incentives for Sustainable Practices

Incentives for sustainable practices in dental waste management and gypsum recycling encompass economic, environmental, and social benefits, each contributing to a holistic approach towards sustainability. Cost savings represent a significant economic incentive, as recycling gypsum diminishes the necessity for new gypsum mining, conserving natural resources and reducing expenses. The versatility of recycled gypsum allows its utilization across various applications, including agricultural products, new drywall production, and other industries, further enhancing cost-effectiveness [15,21]. From an environmental perspective, gypsum recycling holds substantial benefits by mitigating pollution, solid waste generation, and energy and water usage while also curbing waste management costs. Importantly, recycling prevents gypsum from accumulating in landfills, where it can produce toxic gases, thus safeguarding environmental integrity [27].

Social benefits emerge through using recycled gypsum as a fertilizer and soil amendment, fostering sustainability in crop production and enhancing soil health [28]. Furthermore, adherence to specific waste management regulations ensures regulatory compliance for dental practices, thereby averting fines and penalties [27], while also contributing to overall public health by reducing exposure to toxic substances and fostering a cleaner environment [25]. Education and awareness initiatives play a pivotal role, as knowledge dissemination among dental students and professionals regarding effective recycling of dental materials and waste management facilitates the successful implementation of gypsum recycling in dental practices [25]. Moreover, industry growth opportunities abound with the increasing demand for sustainable practices and gypsum recycling, presenting avenues for business expansion within the dental sector [22]. By embracing sustainable practices and promoting gypsum recycling, dental practices realize economic savings and contribute to a more environmentally friendly and responsible approach to waste management, thus embodying a commitment to sustainability across multiple dimensions.

Potential Policy Recommendations for Gypsum Recycling

To promote the circular economy for gypsum products, on-site segregation of gypsum waste emerges as a fundamental best practice [28]. Governments hold the capacity to foster this practice by providing comprehensive guidelines and offering incentives for waste segregation. Encouraging adherence to such protocols can maximize the potential for efficient recycling and reuse of gypsum materials. Furthermore, governments can play a pivotal role in stimulating gypsum waste recycling efforts by providing incentives and promoting the utilization of recycled gypsum in various sectors, including agriculture and construction [27]. By incentivizing recycling initiatives, governments can bolster the market demand for recycled gypsum products, promoting sustainability and resource conservation.

Developing robust regulatory frameworks is paramount in properly disposing of gypsum waste and fostering recycling practices. Governments can enact regulations mandating the separate collection of gypsum waste, delineating requirements for landfill disposal, and promoting recycling endeavors [27]. By instituting such regulations, governments can establish clear guidelines for waste management practices and facilitate compliance within the industry. Raising awareness among dental practices and construction companies regarding the benefits of gypsum recycling and proper waste management practices is imperative. Governments can undertake this by implementing education and outreach programs to disseminate information about the advantages of gypsum recycling and foster a culture of responsible waste management [22]. Moreover, governments can drive innovation in gypsum waste recycling by investing in research initiatives. This entails exploring new recycling technologies, identifying novel applications for recycled gypsum, and enhancing the efficiency of recycling processes [23]. By supporting research and development efforts, governments can facilitate the evolution of gypsum recycling practices, thereby mitigating the environmental impact of gypsum waste and advancing sustainability objectives. These comprehensive policy recommendations promote gypsum recycling initiatives and contribute to reducing environmental degradation stemming from gypsum waste.

Patient and professional awareness

Importance of Educating Patients

Educating patients about the significance of sustainable dentistry and gypsum recycling is important in fostering awareness and advocating for environmentally friendly practices. Patients play a substantial role in the waste generated during dental procedures, underscoring the critical need to comprehend its impact. Firstly, promoting sustainable practices to patients, including gypsum recycling and other environmentally conscious dentistry approaches, can inspire them to actively support dental offices committed to environmental responsibility [31]. By imparting knowledge about sustainable options, patients can make informed choices that align with their values and contribute to environmental conservation efforts. Moreover, raising awareness among patients about the environmental repercussions of dental waste can prompt them to opt for treatment options that minimize waste generation, such as digital impressions and reusable dental products [32]. This informed decision-making empowers patients to actively participate in reducing their ecological footprint within the dental care setting.

Additionally, informed patients are more likely to support gypsum recycling initiatives implemented within dental practices, thereby bolstering the success and efficacy of these programs [33]. Dental offices can cultivate a sense of shared responsibility for environmental preservation by engaging patients as partners in recycling endeavors. Furthermore, dental practices prioritizing patient education on sustainable dentistry and recycling showcase their dedication to environmental stewardship, enhancing their reputation and fostering patient loyalty [34]. By integrating patient education about gypsum recycling and sustainable dentistry into their practice ethos, dental professionals contribute to a more environmentally conscious patient community and play a vital role in safeguarding the planet's health.

Training for Dental Professionals on Sustainable Practices

Training dental professionals on sustainable practices is imperative for advancing eco-friendly dentistry and mitigating the environmental impact of dental care. Various organizations and institutions offer comprehensive training programs and resources tailored to empower dental professionals with the knowledge and skills necessary to embrace sustainability in their practices. One notable resource is the Sustainability in Dentistry Resource Kit developed by Dentsply Sirona, which forms part of its Clinical Educational program, DS Academy. This curriculum offers Continuing Education (CE) credits for completed courses led by experienced dentists and sustainability experts, providing dental professionals with valuable insights into sustainable dentistry practices [35].

Furthermore, a short three-part foundation course on Sustainable Dentistry offers a comprehensive overview of the field for dental care staff. This course equips dental professionals with actionable strategies for promoting sustainability by covering topics such as the importance of sustainability, case studies on 'greening' dental practices, and practical tips for implementing sustainable practices [11]. The FDI World Dental Federation has also developed a Massive Open Online Course (MOOC) on Sustainability in Dentistry, aimed at helping oral health professionals understand the environmental aspects of dental care and integrate sustainable practices into their work [10].

Moreover, ProDental CPD offers the P300 Sustainability in Dentistry Foundation Course, designed to educate dental care professionals and support staff on the impact of dentistry on climate change and ecological degradation. This course empowers participants to implement sustainable practices in their dental offices, contributing to environmental conservation efforts [36]. Dentsply Sirona further enhances educational opportunities with its Sustainable Dentistry Courses & Trainings, providing interactive learning experiences focused on the importance of sustainability, key terms, goals, initiatives, and practical steps for working more sustainably in dental practices [37]. Participation in these training programs equips dental professionals with the knowledge and skills to integrate sustainable practices into their dental offices, thereby contributing to a healthier planet and delivering environmentally conscious care to their patients.

Promoting Sustainable Dentistry in Dental Education

Incorporating sustainable dentistry into dental education is essential for fostering awareness and advocating for environmentally friendly practices. Dental schools and institutions can achieve this by integrating sustainability into their curriculum through courses and training programs. These initiatives cover a range of sustainable dentistry practices, including waste reduction, selecting eco-friendly materials, and promoting oral disease prevention [10, 38, 39]. Additionally, dental professionals can play a vital role in educating their patients on sustainable dentistry practices, emphasizing the importance of waste reduction and oral disease prevention [39]. By empowering patients with knowledge and actionable steps, dental professionals contribute to promoting sustainability within the dental community. Furthermore, dental schools and institutions can establish sustainability committees to spearhead initiatives to promote sustainable practices and foster student involvement [35,40]. These committees serve as platforms for collaboration and innovation, driving the integration of sustainability principles into dental education and practice. By embedding sustainability into dental education, dental professionals enhance their understanding and practices and contribute to the cultivation of an environmentally conscious dental community and a healthier planet.

Future trends and innovations

Emerging Technologies in Gypsum Recycling

The future trajectory of gypsum recycling is marked by several prominent trends and innovations, encompassing technological advancements, increased demand for recycled gypsum, and concerted efforts to tackle associated challenges. Firstly, technological advancements represent challenges and opportunities within the gypsum recycling landscape. While the market for gypsum recycling is poised for growth, recycling companies encounter hurdles in effectively separating hazardous waste and processing recyclable materials. However, ongoing initiatives in research and development, coupled with strategies for managing landfill taxes and incentivizing recycling, present avenues for potential growth [41]. Moreover, a surge in demand for recycled gypsum is anticipated, driven by its myriad applications spanning soil amendment, wastewater treatment, cement additives, and infrastructure development. With burgeoning end-use industries globally, such as the construction sector, and governmental initiatives to bolster infrastructure like highways, the demand for recycled gypsum is expected to skyrocket [41].

Concomitantly, escalating environmental concerns and regulatory measures underscore the intrinsic value of gypsum processing and recycling. As sustainability takes center stage, recycling gypsum emerges as an imperative solution to circumvent disposal challenges, effectively transforming an environmental liability into a lucrative business opportunity [21]. Addressing the logistical aspects of gypsum recycling necessitates attention to source separation and the expansion of recycling infrastructure. As the recycling sector evolves to meet escalating demands, regulators across various states respond to sulfate-related landfill issues, signaling an upsurge in gypsum drywall recycling efforts in the U.S. [22]. Furthermore, the industry is witnessing a noticeable uptick in recycled gypsum waste volumes, sparking discussions on strategies to expedite recycling. This surge in interest is evidenced by the influx of emerging recyclers into the market, underlining the importance of collaboration and concerted industry protection efforts [22]. Looking ahead, the future landscape of gypsum recycling is poised for sustained growth, buoyed by increased demand, technological breakthroughs, and concerted efforts to surmount existing challenges. Collaboration, coupled with proactive industry protection measures and a heightened focus on gypsum recycling, is expected to shape the trajectory of the industry moving forward.

Integration of Sustainable Materials in Prosthodontics

Embracing sustainable materials within prosthodontics holds the promise of reducing the environmental footprint of dental care while fostering a more sustainable and eco-friendly approach to treatment. Digital dentistry stands at the forefront of this movement, offering avenues to minimize waste and energy consumption in dental offices. Technologies such as digital impressions replace traditional molds and materials. At the same time, computer-aided design and manufacturing (CAD/CAM) enable the creation of custom dental prosthetics, thus streamlining processes and reducing resource usage [42]. Furthermore, adopting recycled and biodegradable materials represents a pivotal step toward promoting a circular economy within dental prosthodontics. Utilizing materials like recycled gypsum and biodegradable polymers reduces waste and aligns with sustainable principles, contributing to a greener, more environmentally conscious practice [42].

In tandem with material innovation, implementing reusable and sterilizable impression trays presents a practical solution to curb waste generation. By minimizing reliance on disposable gypsum trays, dental offices can significantly reduce their environmental impact while maintaining high standards of clinical care [39]. Moreover, conservation efforts extend beyond materials to encompass water and energy usage in dental facilities. Implementing water-saving measures and optimizing energy consumption contribute to mitigating the environmental impact of dental care, fostering a culture of sustainability within the profession [10]. Encouraging dental manufacturers to prioritize the development of sustainable materials is paramount. By incentivizing the use of biodegradable and recyclable materials, the industry can drive meaningful change toward a more sustainable future for dental care [10]. Ultimately, by integrating sustainable materials and practices into prosthodontics, dental professionals can play a pivotal role in advancing environmental stewardship within the field. Embracing these initiatives not only benefits patients' health but also contributes to the planet's well-being, forging a path toward a greener, more sustainable future for dental care.

Collaborative Efforts in the Dental Industry

Collaboration within the dental industry is increasingly acknowledged as pivotal for enhancing patient care, fostering innovation, and navigating contemporary challenges in healthcare. The partnership between dental laboratories and offices is a cornerstone for optimizing case acceptance and elevating patient outcomes. Leveraging digital tools and fostering seamless information exchange are key components of this collaboration, which relies on robust, interdependent relationships to ensure the effective delivery of restorative treatments [43]. A paradigm shift is underway in the composition of the oral health care team, emphasizing the inclusion of a broader spectrum of medical professionals. This redefinition advocates for a new care model wherein dentists assume leadership roles within the oral health domain and with other healthcare professions [44]. Opportunities for collaboration abound between the corporate sector and dental education realms. Joint initiatives, enhanced efficiency, and addressing workforce demands are among the potential avenues for collaboration. Closer integration between industry experts and academic faculty, alongside the development of continuing education programs, represents fertile ground for collaborative endeavors [45].

Effective collaboration between dentists and other healthcare providers, notably medical doctors, is imperative for addressing the intricate connections between oral health and overall well-being. By working in tandem, these professionals can ensure comprehensive patient care and confront the systemic implications of oral health on overall health outcomes [46]. Furthermore, the dental industry is undergoing a transformative phase to meet the challenges posed by escalating healthcare costs, heightened market competition, and evolving consumer expectations. Embracing technological advancements, undergoing digital transformation, and fostering enhanced collaboration and communication are pivotal strategies for modernizing the industry and staying ahead of the curve [47]. Collaboration permeates every facet of the dental industry, serving as a linchpin for advancement and adaptation in an ever-evolving healthcare landscape. Whether between laboratories and offices, within interdisciplinary healthcare teams, or across sectors and professions, collaborative efforts are essential for ensuring optimal patient care, driving innovation, and fortifying the dental industry for the challenges of tomorrow.

## Conclusions

This comprehensive review underscores the paramount importance of sustainable dentistry, focusing on the recycling of gypsum products in prosthodontics. The key findings emphasize the feasibility and environmental benefits of adopting recycling techniques for gypsum, shedding light on various methods and successful case studies. As the dental community grapples with the environmental impact of its practices, a resounding call to action emerges. It is imperative for dental professionals, educators, and policymakers to collectively prioritize sustainable dentistry, emphasizing responsible waste management, the use of eco-friendly materials, and the integration of recycling practices in prosthodontics. Looking to the future, the prospects for sustainable dentistry in prosthodontics appear promising, driven by emerging technologies and a growing awareness of the need for eco-conscious oral healthcare. The review envisions a future where the dental community leads the way in implementing innovative and environmentally friendly practices, contributing to a global movement towards a more sustainable and responsible approach to oral health.
